# Concurrent validity of five prediction equations to evaluate fat percentage in a sports group expected to yield high performance from Medellín, Colombia

**DOI:** 10.7705/biomedica.5333

**Published:** 2020-10-19

**Authors:** Ana Lucía López, Juan David Vélez, Angélica María García, Elkin Fernando Arango

**Affiliations:** 1 Instituto de Deportes y Recreación de Medellín, INDER, Medellín, Colombia; 2 Facultad de Ciencias para la Salud, Universidad de Caldas, Manizales, Colombia

**Keywords:** Body composition, nutritional status, anthropometry, child, adolescent, nutrition assessment, adipose tissue, absorptiometry, photon, **c**omposición corporal, estado nutricional, antropometría, niños, adolescentes, evaluación nutricional, tejido adiposo, absorciometría de fotón

## Abstract

**Introduction:**

No equations to predict the body composition of athletes from Medellín expected to have high performance have been constructed and, thus, decisions regarding their training and nutrition plans lack support.

**Objective:**

To calculate the concurrent validity of five prediction equations for fat percentage in a group of athletes from Medell**í**n, Colombia, expected to yield high performance.

**Materials and methods:**

We conducted a cross-sectional analysis to validate diagnostic tests using secondary-source data of athletes under the age of 18 who were part of the “Medellín Team”. The gold standard was dual-energy X-ray densitometry (DEXA). We analyzed the Slaughter, Durnin and Rahaman, Lohman, and Johnston prediction equations, as well as the five-component model. We used the intraclass correlation coefficient to assess the consistency of the methods and the Bland-Altman plot to calculate the average bias and agreement limits of each of the equations.

**Results:**

We included 101 athletes (50,5% of them women). The median age was 14,8 years (IR: 13,0 - 16,0). The concurrent validity was “good/excellent” for the Johnston and the Durnin and Rahaman equations and the five-components model. The Lohman equation overestimated the fat percentage in 12,7 points. All of the equations showed broad agreement limits.

**Conclusions:**

The Durnin and Rahaman and the Johnston equations, as well as the five- component model, can be used to predict the FP in the study population as they showed a “good/excellent” concurrent validity and a low average bias. The equations analyzed have low accuracy, which hinders their use to diagnose the individual fat percentage within this population.

## Abstract (es)

**Introducción:**

La falta de ecuaciones de predicción de la composición corporal de deportistas con expectativas de alto rendimiento en Medellín dificulta la toma de decisiones para su entrenamiento y nutrición.

**Objetivo:**

Calcular la validez concurrente de cinco ecuaciones de predicción del porcentaje de grasa en un grupo de deportistas con expectativas de alto rendimiento en Medellín, Colombia. **Materiales y métodos.** Se hizo un estudio trasversal de validación de pruebas diagnósticas con datos de una fuente secundaria de deportistas menores de 18 años pertenecientes al “Team Medellín”. La densitometría dual de rayos X (DEXA) fue la prueba de referencia. Se analizaron las ecuaciones de predicción de Slaughter, de Durnin y Rahaman, de Lohman y de Johnston, así como el modelo de cinco componentes. Para evaluar la concordancia entre los métodos se utilizó el coeficiente de correlación intraclase y se hicieron análisis de Bland y Altman para calcular el sesgo promedio y los límites de acuerdo de cada una de las ecuaciones.

**Resultados:**

Participaron 101 deportistas (50,5% de ellos mujeres) con una mediana de edad de 14,8 años (RI: 13,0-16,0). La validez concurrente fue “buena-excelente” para las ecuaciones de Johnston, Durnin y Rahaman y el modelo de cinco componentes. La ecuación de Lohman sobreestimó el porcentaje de grasa en 12,7 puntos porcentuales, pero todas mostraron límites de acuerdo amplios.

**Conclusiones:**

En la población del estudio se pueden utilizar las ecuaciones de Durnin y Rahaman, la de Johnston y el modelo de cinco componentes para predecir el porcentaje de grasa, pues su validez concurrente fue “buena-excelente” y el sesgo promedio fue bajo. Las ecuaciones que se estudiaron tienen poca precisión, lo que dificulta utilizarlas para el diagnóstico individual del porcentaje de grasa en dicha población.

Body composition and the changes it undergoes play an important role in athletes’ performance, particularly in those who are in the process of physical development as their physical abilities directly affect their performance and the risk of injuries when practicing sports modalities based on resistance, strength, power, or speed (1-4). It is vital, then, to count with evaluation methods of proven validity and reliability (3-5).

The personnel in charge of athletes who are still developing their abilities or those that are already professionals use doubly indirect methods to measure the body composition: the data derived from measuring skin folds and the information from bioimpedance devices later used to feed prediction formulas specifically adjusted to the population for whom they were developed that may not necessarily be applicable elsewhere, as is the case of athletes from Medellín expected to become high performers (1,2,4).

There are no validated methods to assess the body composition of athletes undergoing training in Medellín, which makes it difficult to get accurate and reliable data to improve decision-making related to their training and nutrition processes. This means these athletes are at disadvantage in their progress towards becoming high-performing athletes compared to those in developed countries (4,6).

Therefore, it is crucial to validate the prediction equations most commonly used to assess athletes’ body composition, such as the Slaughter, Lohman, Johnston, and Durnin and Rahaman equations, and the multicomponent model.

In this context, the purpose of our study was to determine which of these prediction equations designed for children under the age of 18, best suits athletes training in Medellín and get more precise and reliable data to better guide training plans, as well as food interventions adjusted to their environment. The specific goal of the study was to clarify the concurrent validity of five body composition prediction equations in a group of athletes training in the city of Medellín, Colombia.

## Materials and methods

### Type of study

We conducted a cross-sectional quantitative study to validate diagnostic tests.

### Population, sample, and sample design

The population consisted of athletes from Medellín, Colombia, with a high-performance forecast. We used a convenience sample including athletes belonging to the “Team Medellín” (INDER and Medellin Mayor’s Office). The sample size was not calculated, given that almost all of the athletes who are part of this program were included (116 athletes).

### Selection criteria

We included athletes who met the following criteria: Being part of the “Team Medellín) during 2018 and 2019 (first four months), and consent by both the athletes and their legal advisors (legal representatives) to use the existing data recorded in their nutritional assessment during the mentioned years. We excluded those with health disorders that could alter their body composition characteristics, such as malignant conditions, thyroid disorders, or other endocrine disorders, as well as 18-year old athletes or older.

### Bias control

We used secondary-source data collected by three nutrition and dietetics professionals trained in anthropometry (International Society for the Advancement of Kinanthropometry) level 2 certification to minimize intra and inter-observer variability. Moreover, we used calibrated and validated

equipment to assess the body composition of all athletes following a protocol set for this purpose. Selection criteria were strictly verified. Besides, the database was subject to debugging and quality control. Extreme values or outliers were sought as they can affect the means of quantitative variables and, therefore, the results of the parametric statistical tests. Missing data and typing errors were also looked for and corrected when necessary. Missing data was addressed through multiple imputation, a process that did not need to be carried out.

### Instruments and information collection

As already mentioned, we used secondary-source data collected during the nutritional assessments conducted in 2018 and the first four months of 2019. The age was calculated using the date of birth; gender was determined according to the primary sexual characteristics; socioeconomic stratum was obtained from the participants’ self-report and then reclassified as follows:

1) Low, corresponding to 1 and 2 strata; 2) middle, those residing in 3 and 4 strata city locations, and 3) high, those residing in 4 and 5 strata locations.

The type of sport was classified according to the intensity of resistance, its duration, and the predominant metabolic pathway during its practice as follows:

1. Explosive resistance (maximal intensity and duration close to 6 seconds; use of phosphagens);

2. high-intensity resistance (less than maximal high intensity and duration of >6 seconds and 1 minute; glycolysis), and

3. intense resistance strength (actions during more than 1 minute mainly using oxidative phosphorylation) (7).

Weight was defined as the amount of body mass measured in kilograms with a 150 kg-capacity and 100 g-accuracy Seca 803 scale™; height was measured as the length between the lowest part of the heel and the highest part of the skull with the athlete standing at the end of inhalation using a Charm HM200P™ stadiometer with a range between 14 and 205 cm and an accuracy of 1 mm; the body mass index (BMI) was calculated using the Quetelet formula [BMI = weight (kg) / height (m^2^)]; specific sports life-time was defined as the years of practicing the current specific sport; weekly training time was the amount of time devoted to training and competing each week; schooling was calculated based on the years of formal education that each athlete acknowledged to have received at the time of the body composition assessment.

The gold standard to assess body composition were the values obtained using a dual-energy x-ray absorptiometry (DEXA) device (General Electric Lunar Prodigy™), which measures the bone mineral mass and soft tissues (lean and fat masses) separately, is non-invasive, and generates low levels of radiation (equivalent to one day or less of solar radiation). It offers information on three components: fat, fat-free mass, and bone mineral content (1,5,6,8). Test-retest reliability of DEXA devices shows variation coefficients (% VC) suitable for assessing fat-free mass of 0.8% (SD=0.4), fat mass of 2.6% (SD=1.2), and bone mineral density of 1.0% (SD=0,9) (4).

A Harpenden caliper was used to measure the skin folds by applying a pressure of 10 g/mm^2^ regardless of the thickness of the fold; its accuracy level is 99%, its precision is 0.2 mm, and the measuring range is 0 to 80 mm.

To assess body composition (fat percentage and fat-free mass), we used the following prediction equations, which are considered doubly indirect methods (table 1) (8-12).

**Table 1:** Equations for the prediction of fat percentage and adiposity within the athletes under 18

**Equation**	**Variables**	**Population**
Slaughter (8)	• Folds: Tricipital and calf	It dates back to 1988; it is recommended for children between the ages of 8 and 17; it was built using a sample of 59 African-American and Caucasian people (30 boys and 29 girls) from Illinois and Arizona (USA).
Durnin and Ramahan (9)	• Folds: Triceps, biceps, subscapular, and suprailiac	It was developed in 1967 to get the body density and then calculate the fat percentage using the SIRI equation. The sample consisted of English people (Great Britain) as follows: 38 girls participated in the formula for girls with ages ranging between 13,2 and 16,4 years; 48 boys participated in the formula for boys with ages ranging between 12,7 and 15,7 years.
Lohman (10)	• Age • Sex • Body weight (kg) • Folds: Triceps and suprailiac	This equation was obtained using a sample of 39 boys and 59 girls, all of American Indian origin, from Arizona, USA.
Johnston (11^)^)	• Folds: Triceps, biceps, subscapular, and suprailiac	Equations created in 1988 to calculate body composition, and then obtain the fat percentage using the SIRI equation. The sample consisted of 168 girls and 140 boys from Canada with ages ranging from 8 to 14.
Five-component model (12)	• Body weight (kg) • Height (cm) • Height while sitting (cm) • Perimeters: Head, relaxed arm, forearm, thigh, calf, rib cage, waist • Diameters: Biacromial, biiliocrestal, humerus, femur, anteroposterior and transverse of the rib cage • Folds: Triceps, subscapular, supraspinal, abdominal, thigh, calf	The five-component method was proposed in 1982 for a sample of 1,669 people of both sexes: university students, school students, and athletes, with ages ranging between 6 and 77. This method had an excellent correlation with the dissection of corpses (0,987).

The proportion of fat mass calculated using the five-component model was converted to FP using the following procedure:

1. The adiposity percentage (% Adip) was calculated using the five-component method;

2. Fat mass was obtained by multiplying each participant’s proportion of adiposity by his/her weight;

3. The lipid fraction of adiposity was obtained using the formula proposed in 1994 by Martin, *et al* (13): LF = 0.327 + (0.0124 x % Adip);

4. Lipid mass of adiposity was calculated by multiplying the fat mass by lipid fraction, and

5. The weight fat percentage was obtained by dividing the lipid mass of the adiposity by the body weight, which was then multiplied by 100.

### Assessments

The anthropometric assessments were carried out in doctor’s offices authorized by the sectional health service of Antioquia from 8 a.m. to 5 p.m. Each assessment session took approximately 45 minutes and was done by three nutrition and dietary professionals with ISAK 2 certification; every athlete was

accompanied by an adult. The participants wore comfortable clothes that allowed easy access to the anatomical sites to be measured that were then marked with a black dermal pencil on the right side of the body; out of the 23 sites included in the ISAK protocol, six were not required. Finally, we registered the data regarding the skin folds (10 points), the perimeters (4 points), the lengths (9 sites), and the diameters (11 points).

After the anthropometric assessment, the participants were assessed with the DEXA device for the total body fat percentage and the fat-free mass by three nutrition and dietary professionals trained for the test following the device manufacturer’s manual. Before the assessments, the equipment was calibrated and the athletes’ hydration status was not measured, although they were advised to hydrate constantly and not to engage in strenuous physical activity for the previous 24 hours.

### Ethical aspects

Parents and athletes gave written consent for the use of the data from their nutritional histories. The CES University Ethics in Human Research Committee (Certificate 139, August 16, 2019) approved the study, which adhered to the guidelines of the Declaration of Helsinki and Resolution 8430 of 1993 issued by the Colombian Ministry of Health. Participants’ privacy was safeguarded, as well as the confidentiality of their data used exclusively for scientific and academic purposes. Only the researchers had access

through the password to the database and the information was stored without participants’ names or identification.

### Statistics analysis

We used a Shapiro Wilk test to establish the distribution of quantitative variables; those with a normal distribution were summarized with means and standard deviations (SD), for those that did not we used medians and

interquartile ranges (IR), and qualitative variables were expressed in proportions.

We used the intraclass correlation coefficient (ICC) to assess the concurrent validity of each of the prediction formulas assuming values below 0,40 as having “poor” concordance, those between 0,41 and 0,75 as “moderate” and over 0,75 as “good/excellent”.

We used the Bland-Altman plot method to analyze the concordance between measurement methods calculated with the means and SD of the differences in the fat percentage measurements (DEXA - prediction formulas) from the Lohman, Slaughter, Durnin and Rahaman, and Johnston equations and the five-component model. We also obtained measurement biases and limits of agreement.

Statistical analyzes were done with the SPSS™ software, version 21, with 95% reliability and an alpha error (statistical significance) of less than 5% (p<0,05). We assessed the validity of each of the prediction equations according to the sex.

## Results

### Participants’ characteristics

Data was collected during the second semester of 2018 and the first semester of 2019. Participation included 101 athletes of whom 50,5%

(n=51) were women; the median age was 14,8 years old (IR=13,0-16,0); the schooling median was 10,0 years (RI=8,0-11,0); the average sports life was 6,5 years (SD=2,2); no statistically significant differences were found when stratifying these variables by sex (table 2).

**Table 2:** Socio-demographic and anthropometric characteristics of participants (n=101)

**Variables**		**Women**	**Men**		**All**	
Proportion of participants	51	(50.5%)	50 (49.5%)		101 (100%)	
Age (years)*	14.8	(13.0 to 16.0)	14.9 (13.0 to 16.0)		14.8 (13.0 to 16.0)	
Schooling (years)*	10.0	(8.0 to 11.0)	9.5 (8.0 to 11.0)		10.0 (8.0 to 11.0)	
Socio-economic stratum: Low	8	(15.6%)	11 (22.0%)		18.8%	
Medium	28	(54.9%)	24 (48.0%)		51.5%	
High	14	(27.4%)	15 (30.0%)		28.7%	
Sports life (years)**	6.2	(2.2)	6.7 (2.2)		6.5 (2.2)	
Type of sport: Resistance	17	(33.0%)	16 (32.0%)		33 (32.7%)	
High-intensity	31	(60.8%)	32 (64%)		63 (62.4%)	
Long-duration	1	(2.0%)	0 (0.0%)		1 (1.0%)	
Other	2	(3.9%)	2 (4.0%)		4 (4.0%)	
Weight (kg)**	50.9	(8.6)	56.1 (13.8)		53.5 (11.7)	
Height (cm)* 160.5 (153.1 to 164.6) 168.3 (151.8 to 173.6) 162.0 (152.5 to 169.8)						
BMI (kg/m2)**	20.1	(2.3)	20.5	(2.5)	20.3	(2.4)
% fat (DEXA)*	27.3	(24.2 to 30.6)	19.2	(15.2 to 22.2)	23.0	(17.7 to 28.2)
% fat (Slaughter)*	20.7	(18.0 to 23.2)	12.9	(11.6 to 16.9)	17.5	(12.5 to 21.7)
% fat (Durnin and Rahaman)*	26.8	(24.6 to 29.3)	17.7	(15.4 to 20.2)	22.1	(17.4 to 27.1)
% fat (Johnston)*	23.7	(21.5 to 26.2)	15.8	(13.4 to 18.5)	19.8	(15.5 to 24.6)
% fat (Lohman)*	37.9	(34.9 to 39.6)	34.2	(31.6 to 37.4)	35.9	(32.8 to 38.9)
% adiposity (Five-component model)*	34.3	(29.7 to 37.8)	27.4	(25.4 to 30.9)	30.5	(26.9 to 35.4)

BMI: Body mass index; DEXA: Dual-energy x-ray absorptiometry

* Values provided in averages and interquartile ranges

** Values provided in means and standard deviations

One of every two (51,5%) participants was classified in the middle socioeconomic stratum and one fifth (18,8%) of them in the low one. A third (32,7%) practiced power sports and two out of three athletes (60,8%) were involved in high-intensity sports (table 2).

Weight (mean 56,1 kg vs. 50,9 kg) and height (168,3 cm vs. 160,5 cm) were higher in men, while the fat percentage measured with the prediction equations and DEXA was higher in women (median: 27,3% vs. 19,2%), as well as the percentage of adiposity measured with the five-component model (table 2).

### Concurrent validity

Regarding the concurrent validity of the prediction equations of fat percentage vs. DEXA, we found that those with “good/excellent” ICC corresponded to the Johnston (0,833; IC95% 0,290 to 0,935), Durnin and Rahaman (0,912; IC95% 0,867 to 0,941), and the five-component (0,853; IC95% 0,783 to 0,901) equations; those with “moderate” ICC corresponded to the Slaughter (0,741; IC95% -0,186 to 0,921) equation, and those with “poor” ICC corresponded to the Lohman (0,248; IC95% -0,130 to 0,590) equation. These results changed very little when we analyzed them by sex. The 95% confidence interval (IC95%) for the Lohman (all, women, and men), Slaughter (all, women, and men) and Johnston (women) equations had negative lower limits and positive upper limits (table 3).

**Table 3:** Concurrent validity of the body fat percentage prediction equations

**Gold** **Standard**	**Prediction equation**	**Sample**	**ICC**	**CI 95%**
DEXA	Slaughter	Women	0.618	-0.161 to 0.880
		Men	0.666	-0.216 to 0.888
		All	0.741	-0.186 to 0.921
	Durnin and Ramahan	Women	0.874	0.779 to 0.928
		Men	0.795	0.640 to 0.884
		All	0.912	0.867 to 0.941
	Lohman	Women	0.341	-0.094 to 0.715
		Men	0.082	-0.081 to 0.298
		All	0.248	-0.130 to 0.590
	Johnston	Women	0.736	-0.111 to 0.908
		Men	0.732	0.285 to 0.878
		All	0.833	0.290 to 0.935
	Five-component model (% fat)	Women	0.770	0.593 to 0.870
		Men	0.800	0.648 to 0.887
		All	0.853	0.783 to 0.901

DEXA: Dual-energy x-ray absorptiometry; ICC: Intraclass correlation coefficient

When we assessed the concurrent validity of each of the prediction equations for the fat percentage versus the DEXA value obtained using the Bland-

**Figure 1:**
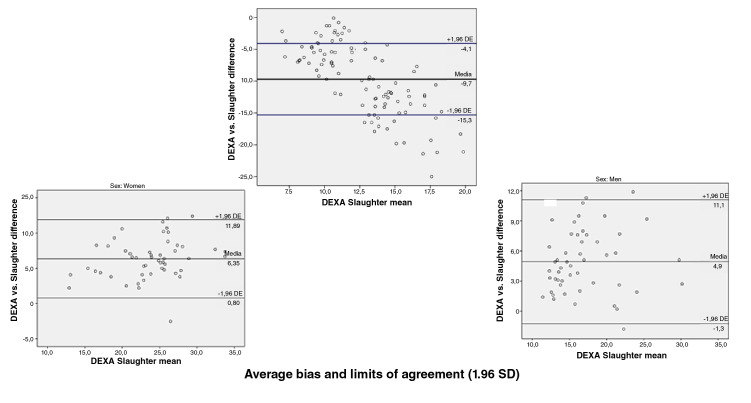
Bland and Altman graphs - Fat percentage concordance analysis: DEXA vs. Slaughter equation

**Figure 2:**
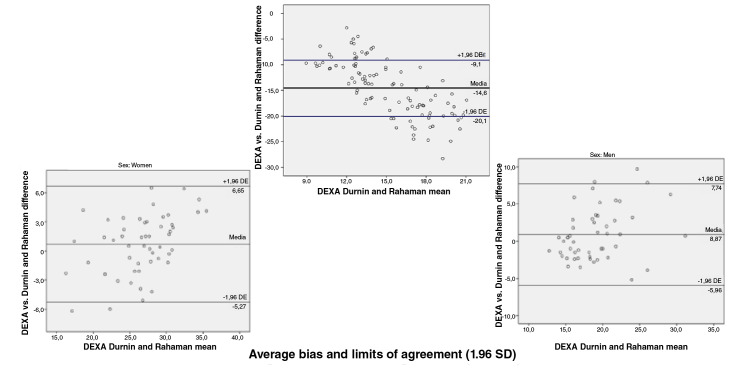
Bland and Altman graphs - Fat percentage concordance analysis: DEXA vs. Durnin and Rahaman equation

**Figure 3:**
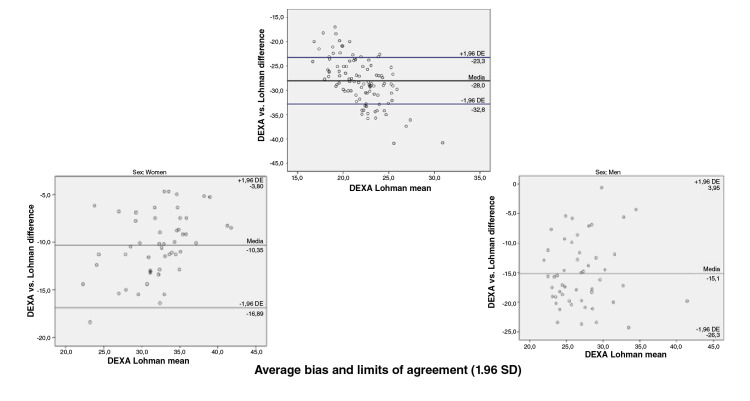
Bland and Altman graphs - Fat percentage concordance analysis: DEXA vs. Lohman equation

**Figure 4:**
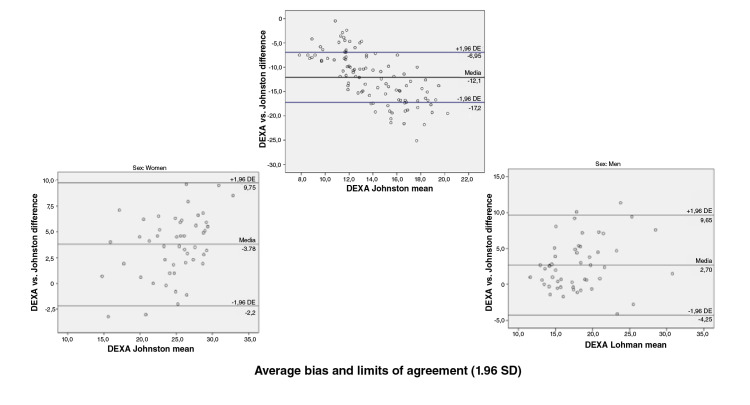
Bland and Altman graphs - Fat percentage concordance analysis: DEXA vs. Johnston equation

**Figure 5:**
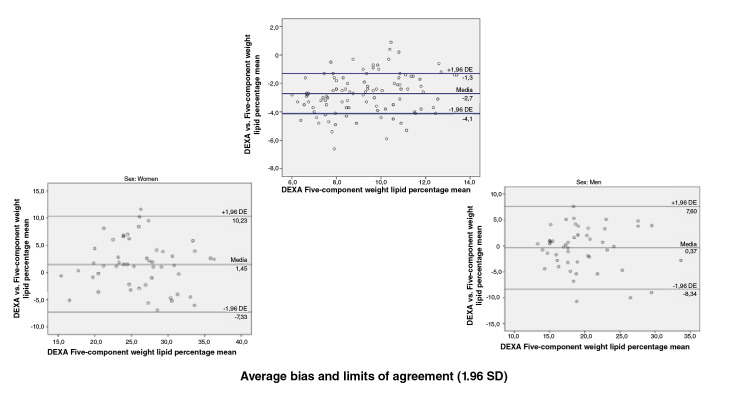
Bland and Altman graphs - Fat percentage concordance analysis: DEXA vs. five component equation Average bias and limits of agreement (1.96 SD)

Altman plot method we found the following: Compared with the DEXA values, the Slaughter, Durnin and Rahaman, Johnston, and five-component model prediction equations underestimated the fat percentage with values ranging in average between 0,6 and 5,6 percentage points; the highest error was that of the Slaughter equation and the lowest that of the five-component model, and the Lohman equation overestimated the fat percentage by 12,7 percentage points. When discerning by sex, a greater bias was found for women with the Slaughter (6,4 vs. 4,9), Johnston (3,8 vs. 2,7) and five-component (1,5 vs. -0,4) equations; likewise, the error was greater in men when using the Lohman (-15,1 vs. -10,4) and Durnin ans Rahaman equations (0,9 vs. 0,7) (figures 1 to 5).

Concordance limits were extensive for all the equations (Slaughter: -0,4 - 11,6; Durnin and Rahaman: -5,6 - 7,2; Lohman: -22,9 - -2,5; Johnston: -3,3 - 9,8; five-component model: -7,98 - 9,1). On the other hand, only a low proportion of the equations data was outside the limits of agreement (figures 1 to 5).

## Discussion

We found that compared to the DEXA values the concurrent validity for the Durnin and Rahaman, Johnston, and five-component equations was “good/ excellent”; for the Slaughter equation it was “moderate”, and for the Lohman equation, “poor”, with no significant changes in these values after stratifying by sex, except for the Lohman equation, whose values were almost 5 percentage points lower for men in average.

All equations underestimated the fat percentage in average percentage points ranging between 0,6 and 5,6 and had extensive concordance limits, except the Lohman equation, which overestimated it.

### Validity of the Slaughter equation

The Slaughter equation showed an ICC of 0,741 (IC95%: -0,186 to 0,921), which varied very little when discerning by sex. These results are not comparable to those from other studies given that in them, the Pearson or Spearman correlation coefficients were used to assess the concurrent validity of this equation vs. DEXA (14,15).

On the other hand, the average bias reached 5,6 percentage points with limits of agreement between -0,4 and 11,6, which was higher than the one reported in two Spanish studies: one among 98 soccer players of both sexes and an average age of 13,4 (SD=0.6) with a bias of 3,3 percentage points (limits of agreement: -2,9 to 9,5) (16) and the other among 88 swimmers of both sexes with an average age of 14,3 (SD=1,84) and a bias of 4,1 percentage points (limits of agreement: -2,2 to 10,4) (17). These results agree with those by Garcia, *et al.* in a group of Chilean soccer players (average age=19,9; SD=1,3) with a lower average bias (-1,3 percentage points) and narrower limits of agreement (-6,1 to 3,5) (15).

Some studies on the validity of the Slaughter equation assessed by comparing it to DEXA among Latin American (Colombia and Chile), Spanish, and African non-athletes under the age of 18 reported concordance results with the Bland-Altman method consistent with those from our research, i.e., fat percentage underestimation with extensive limits of agreement (17,18), which are also similar to those reported in a sample of swimmers in Spain (17).

### Validity of the Durnin and Rahaman equation

This equation showed a “good/excellent” concurrent validity, which was maintained when we assessed the results by sex. These values are difficult to compare with other studies where correlation was calculated but not concordance (17).

In a study conducted among swimmers under the age of 18 comparing the Durnin and Rahaman equation with DEXA, the average bias was -0,46 percentage points (17), lower to the bias we found of 0,8 percentage points in men and 0,7 in women; moreover, these values were close to those reported by Rodríguez, *et al.* in 2005 in a sample of non-athletic adolescents (men:-1,34; women: 0,0), but the limits of agreement were extensive in all three studies (17,18).

### Validity of the Lohman equation

This equation was the only one showing a “poor” concurrent validity especially in men. Compared to DEXA, it overestimated the fat percentage largely (average percentage points for women: -10,4; men: -15,1; all -12,7) with rather extensive limits of agreement. We found no research comparing the Lohman equation to DEXA for fat percentage estimation in people under 18, or in athletes, to contrast our results.

### Validity of the Johnston equation

The Johnston equation had a “good/excellent” concurrent validity (ICC: 0,833; IC95%: 0,290 to 0,935), which was reduced to “moderate” when we stratified by sex (ICC for women: 0,736; for men: 0,732). We found no studies assessing the equation with the ICC; some used the Pearson correlation coefficient to assess the relationship between the variables, but they did not take into account the concordance.

On the other hand, in the Spanish study among soccer players under the age of 18, the average bias for this equation was 2,3 percentage points (limits of agreement: -2,9 to 7,6) while in our study this error was greater: 3,3 percentage points (limits of agreement: -3,3 to 9,8) (16). Similarly, in a study among Spanish men and women with an average age of 15,3 (SD=1,3), the average bias was 2,4 percentage points in women and -1,1 in men, lower than the values found in the current study (women: 3,8; men: 2,7), but the limits of agreement were extensive in both studies (18).

### Validity of the five-component model

The adiposity values measured with this method were converted to fat percentage using a formula that takes into account the lipid fraction of each participant resulting in a “good/excellent” concurrent validity, which was maintained when we disaggregated by sex (13). Using the Bland-Altman method, the average bias was 0,6 percentage points, overestimating the fat percentage in 0,4 points in men and 1,5 points in women. We found no studies using the same procedure we used, nor any that resorted to the ICC or the Bland-Altman analysis as statistical tests to calculate concurrent validity (concordance), average bias, and limits of agreement, so it was not possible to compare our results.

Currently, DEXA is under consideration as a “gold standard” to assess body composition in humans given that it is an indirect method with biases as compared with the only known direct method, which is the dissection of corpses. On the other hand, the manufacturers of DEXA devices have not standardized this technology and there are differences among the models of this very same brand and the software they use, which questions the consistency of results and hinders in vivo estimates of body composition in people (19,20).

Besides, it is known that body composition prediction equations are specific to the population for whom they were developed (20,21). None of the equations we evaluated were developed for Colombians or for athletes expected to be high-performers, which may partly explain the lack of accuracy of their results and the high average bias when compared to DEXA, especially in the case of the Slaughter and Lohman equations, and to a lesser extent, the Johnston’s.

On the other hand, some equations use the prediction of body density as an initial step and, then, they use such value to calculate the fat percentage, which can lead to bias due to the assumptions regarding body density, i.e., lean mass: 1,1 g/cm^3^ and fat mass: 0,9 g/cm^3^ without considering individual variation in the proportions and densities of human body tissues. In that same sense, when the skin folds are used to predict the fat percentage, it is assumed that there is a constant compressibility of the skin and subcutaneous fat and that the thickness of the skin is not variable, but it is well known that the thickness of the skin varies within a population depending on characteristics such as age and sex, which modify the compressibility of the said tissue and leads to measurement errors. Additionally, the relative distribution of body fat is not constant within a population and the proportion of internal fat vs. external fat is not fixed, which can increase measurement errors and, therefore, in body composition predictions (22).

### Study strengths and limitations

A strength was the use of DEXA as a gold standard as its accuracy and reliability are good to assess body composition in children under the age of 18, as well as the use of robust statistical tests (ICC and the Bland-Altman plots) to calculate the concordance and accuracy of the values resulting from the prediction equations studied. Likewise, we used a correction formula that takes the lipid fraction and transforms it to fat percentage in the five- component model to assesses body fat and make it comparable to the DEXA values.

There were some limitations too: the use of secondary source data, which may have introduced an information bias in the final results; furthermore, anthropometric assessments and densitometries were not performed on the same day, which could lead to changes in body composition measurements during that period altering the true concurrent validity of the prediction equations included in the study.

By using DEXA as a gold standard to evaluate the fat percentage in Medellín athletes of both sexes under the age of 18 expected to have high athletic performance, the Durnin and Rahaman, Johnston, and five- component model prediction equations showed a “good/excellent” concurrent validity while the Slaugther equation had a “moderate” one and the Lohman equation a “poor” one.

Compared to the DEXA values, the prediction equations underestimated the fat percentage (average bias in percentage points: five-component model: 0,6; Durnin & Rahaman: 0,8; Johnston: 3,3; Slaughter: 5,6) while the Lohman equation overestimated it (average bias: -12,7 percentage points). The accuracy of the equations was low, which is reflected in the extensive limits of agreement found for each of them.

Our results have several practical implications. First, we recommend using the five-component model converting adiposity to fat percentage and the Durnin and Rahaman equation to predict this percentage in Medellín sports groups of both sexes under the age of 18 with high-performance expectations because they showed lower average biases when compared to the results yielded by DEXA and a “good/excellent” concurrent validity.

None of the equations had an acceptable accuracy, which became evident in the extensive limits of agreement found in all of them. This hinders the use of the data yielded in individual athletes as it can lead to errors in decision- making in terms of the athletes’ training and nourishing plans aimed at optimizing their health and sports performance.

We found no studies validating the prediction equations for body composition compared to DEXA in athletes under the age of 18 in Colombia and the fact is that they are also scarce in the world. Therefore, it has been proposed to develop specific equations for the country’s population of athletes at national, regional, and local levels differentiating, if possible, by sport discipline, age, and sex to optimize athletes’ body composition measurements and, thus, adjust their training and nourishing plans.
